# Psychometric properties of the short form of the Stroke Impact Scale in German-speaking stroke survivors

**DOI:** 10.1186/s12955-021-01826-5

**Published:** 2021-07-31

**Authors:** Anna Coppers, Jens Carsten Möller, Detlef Marks

**Affiliations:** 1 Center for Neurological Rehabilitation, Hauptstrasse 2, 8588 Zihlschlacht, Switzerland; 2 Department of Neurology, Philipps University, Baldingerstr, 35043 Marburg, Germany

**Keywords:** Short-form Stroke Impact Scale, Quality of life, Stroke, Inpatient rehabilitation, Psychometric properties

## Abstract

**Background:**

The short form of the Stroke Impact Scale (SF-SIS) consists of eight questions and provides an overall index of health-related quality of life after stroke. The goal of the study was the evaluation of construct validity, reliability and responsiveness of the SF-SIS for the use in German-speaking stroke patients in rehabilitation.

**Methods:**

The SF-SIS, the Stroke Impact Scale 2.0 (SIS 2.0), EQ-5D-5L, National Institutes of Health Stroke Scale (NIHSS) and de Morton Mobility Index were assessed in 150 inpatients after stroke, with a second measurement two weeks later for the analyses of responsiveness. In 55 participants, the test–retest-reliability was assessed one week after the first measurement. The study was designed following the recommendations of the COSMIN initiative.

**Results:**

The correlations of the SF-SIS with the SIS 2.0 (ρ = 0.90), as well as the EQ-5D-5L (ρ = 0.79) were high, as expected. There was adequate discriminatory ability of the SF-SIS index between patients who were less and more severely affected by stroke, as assessed by the NIHSS. Exploratory factor analysis indicated a two-factor structure of the SF-SIS explaining 59.9% of the total variance, providing better model fit in the confirmatory factor analysis than the one-factorial structure. Analyses of test–retest-reliability showed an intraclass correlation coefficient of 0.88 (95% CI 0.75–0.94). Hypotheses concerning responsiveness were not confirmed due to lower correlations between the assessments change scores.

**Conclusion:**

Results of this analysis of the SF-SIS’s psychometric properties are matching with the validity analysis of the English original version, confirming the high correlations with the Stroke Impact Scale and the EQ-5D-5L. Examination of structural validity did not confirm the presumed unidimensionality of the scale and found evidence of an underlying two-factor solution with a physical and cognitive domain. Sufficient test–retest reliability and internal consistency were found. In addition, this study provides first results for the responsiveness of the German version.

*Trial registration* The study was registered at the German Clinical Trials Register. Trial registration number: DRKS00011933, date of registration: 07.04.2017

## Background

Stroke is a drastic experience for those affected and their environment and often results in permanent reduction in quality of life (QoL) [1, 2]. The greatest changes in QoL after a stroke can be recorded in the first six months and over the rehabilitation stay [3, 4]. In various chronic diseases, the self-assessment of the health-related quality of life (HRQoL) by patients is moving into focus as a relevant target variable to be investigated and influenced [3]. Therefore, specific and sensitive instruments are needed to assess the HRQoL of patients [5].

The Stroke Impact Scale (SIS) was developed in consensus with patients and caregivers to measure the HRQoL after a stroke [6]. Answering all 59 questions in the SIS 3.0 takes 15–20 min and can be time consuming and might be burdensome for those affected [7, 8]. A possible alternative is the short form of the Stroke Impact Scale (SF-SIS) [8]. Jenkinson et al. determined by means of factor analysis that all eight dimensions of the SIS 3.0 can be combined to an overall index. They found a high levels of internal consistency of the eight domains on the SIS 3.0, which suggested some item redundancy [8]. Therefore, they investigated whether single items chosen from each dimension could replicate results on the Physical Function Domain and the SIS Index. For the derivation of the items of the SF-SIS they selected the most highly correlated item from each dimension to the total score of the contributing dimension [8]. After creating this pilot version, a larger dataset from the Virtual International Stroke Trial Archive (VISTA) was used to repeat this analysis for the acute and rehabilitation settings and compare the datasets. The final SF-SIS was created out of the two pilot versions and the consent of a focus group with health professionals in the field of stroke care, stroke patients and their caregivers. The items were selected, that were favored in ≥ 2 of these three data sources [9]. An index (0–100) enables an indication of the “overall” HRQoL across all eight dimensions [9]. Because of its shortened length, the SF-SIS should be less susceptible to missing values [8]. For the final version of the SF-SIS convergent and discriminative validity were assessed using the VISTA dataset, for English-speaking stroke survivors in the acute and rehabilitation setting [9].

In their review, Harrison et al. [10] demand for instruments measuring QoL after stroke to be subjected to the same critical review of quality criteria as other scales. In the present study, the recommendations of an international Delphi study of the Consensus-based Standards for the selection of health Measurement Instruments (COSMIN) initiative are followed. COSMIN developed a checklist to assess the methodological quality of single studies on measurement properties of patient-reported outcome measures, which can also be used as a basis for planning studies on quality criteria [11, 12].

In German, only the older version 2.0 of the SIS has been validated so far in a cross-sectional survey for patients with stroke [5]. This study aimed to investigate the construct validity, reliability and responsiveness of the SF-SIS. The German version of the SF-SIS was conducted in stroke survivors over their stay at inpatient rehabilitation. A better understanding of the short versions’ measurement properties can provide a more practical assessment of the HRQoL after stroke by means of a quick-to-collect instrument. This could be a less burdensome alternative to the SIS for use not only in clinical studies but also in everyday clinical practice.

## Materials and methods

### Design and participants

In this prospective psychometric study patients aged ≥ 18 years with stroke [ICD10 (I60–I64)] were recruited consecutively from April 2017 to April 2018 until the predefined sample size of n = 150 was reached. Based on the recommendations of the COSMIN initiative, a minimum size of n ≥ 100 was assumed [11]. With expected drop-outs over the study period and missing data, a target size of 150 patients was set. Recruitment took place at the Rehaklinik Zihlschlacht, a clinic for neurological inpatient rehabilitation in Switzerland. Patients unable to understand the German study instructions and questions as judged by the investigator were excluded. The same investigator, a physical therapist with 3 years of working experience, carried out all tests and interviews.

The first appointment (T1) took place two weeks after admission in the patient’s room. This appointment was chosen, because the questions of the SF-SIS and SIS 2.0 refer to the last days, one and two weeks and the patients should have the possibility to rate their HRQoL experienced at the rehabilitation setting [9]. The SF-SIS, SIS 2.0 and EQ-5D-5L were collected in interview form [5, 9, 13]. Stroke severity was assessed with the National Institutes of Health Stroke Scale (NIHSS) and the mobility status was determined using the de Morton Mobility Index (DEMMI) [14, 15]. In a subsample of at least 50 patients, the SF-SIS was reassessed one week after the first measurement point (T2). Patients were asked whether they had experienced any changes in QoL since the last appointment. If yes, they were requested to indicate the extent of this change on a Global Rating of Change (GRC) scale. At least two weeks after the first measurement, before leaving the clinic, all assessments were repeated (T3). Again, patients were asked whether they had experienced changes in QoL since the first measurement and requested to quantify these changes on the GRC scale.

The order of the assessments’ conduction was varied with a change between the long and short-version after 50% of the patients in order to rule out systematic bias in a questionnaire due to possible learning effects. The EQ-5D-5L was collected between the two questionnaires so that they did not follow one another directly. DEMMI and NIHSS were also randomly performed at the beginning or end of data collection to prevent possible systematic fatigue effects. For repeated surveys of test–retest reliability, as well as responsiveness, the tests were always carried out under the same spatial conditions and by the study leader.

### Assessments

#### Stroke Impact Scale 2.0 (SIS 2.0)

The Stroke Impact Scale 2.0 (SIS 2.0) covers eight domains: strength, hand function, everyday life, mobility, communication, emotions, memory and participation with a total of 64 items (German version 2.0) [5]. Each item can be rated on a 1–5 point Likert scale. Depending on the question, it is possible to indicate how often an occurrence was observed in everyday life (none of the time to all of the time), how difficult a task is (not difficult at all to could not do at all) or how much strength the person has (a lot of strength to no strength at all). The raw values for each dimension are converted into a final score of 0–100, whereby a high value indicates fewer restrictions. Additionally, the patient should indicate the recovery from the stroke on a visual analog scale (VAS) [5].

#### Short form of the Stroke Impact Scale (SF-SIS)

The eight items determined from the SIS 3.0 for the SF-SIS by MacIsaac et al. [9] were presented in Table 3 of their derivation and validation study: dimension 1 item (c), (2f), (3d), (4e), (5h), (6c), (7e) and (8b). Due to the existence of the validated German version SIS 2.0 these items were available in German language [5]. For this reason, we refrained from a renewed process of translation and intercultural adaptation of these eight questions. As with the SIS 2.0, the rating is based on a 5-point Likert scale (1–5 points). The raw sum score of the eight questions with a range from 8 to 40 points is converted into an interval-scaled total index of 0–100 points, the SF-SIS index. Higher scores indicate a better quality of life [8, 9].

#### EQ-5D-5L

The EQ-5D-5L is a generic measurement instrument for recording HRQoL and has already been validated for use in patients after stroke [13]. The EQ-5D-5L consists of five items covering the following aspects: mobility, self-sufficiency, everyday activities, pain/physical complaints and anxiety [16]. The German version (country: Switzerland, paper version, 2011) with five answer levels per item is used. The answer levels are reaching from no limitations (5 points) to full restriction (1 point) in the five domains. In this work we calculated the Level Sum Score of the EQ-5D-5L (range 0–100, higher scores indicate a better HRQoL) [16]. In addition, the current state of health is recorded on the EQ VAS [16].

#### National Institutes of Health Stroke Scale (NIHSS)

The National Institutes of Health Stroke Scale (NIHSS) measures the severity of symptoms that can be associated with stroke and is used as a quantitative tool to measure neurological deficits after stroke [14]. It consists of 15 items, each rated on an ordinal scale with a total score of 0–42 points (0 = no deficits). Previous investigations found relations between the health-related quality of life and the severity of the stroke, measured with the NIHSS [17].

#### de Morton Mobility Index (DEMMI)

The de Morton Mobility Index (DEMMI) is a mobility assessment that evaluates the performance of 15 everyday activities in five categories (bed, chair, static balance, walking and dynamic balance). Using a conversion table, the determined DEMMI raw value (0–19 points; ordinal scale level) can be converted into the final DEMMI score with higher scores indicating better mobility (0–100 points; interval scale level) [18]. The German version of the DEMMI was administered [19].

#### ***Global Rating of Change (GRC) S***cale

For the self-assessment of a change in QoL by the participants, they were asked if they perceived a change in QoL compared to the last measurement point. In the case of a perceived change, they were asked to rate this change on a Global Rating of Change (GRC) scale with eleven points reaching from: a little, somewhat, moderately, a lot and very much better/worse (range − 5 to + 5). GRC scales of seven to eleven points appear to offer the best compromise between patient preference, adequate discriminative ability, and test–retest reliability [20].

### Construct validity

Construct validity describes the degree to which the scores of an instrument are consistent with hypotheses based on the assumption that the instrument validly measures the construct to be measured [21].

Structural validity, as an aspect of construct validity, describes the degree to which the scores of a patient reported outcome measure are an adequate reflection of the dimensionality of the construct to be measured [21]. Principal component analysis of the eight items of the SF-SIS’s pilot version produced a single factor accounting for 57.25% of the variance with high internal consistency reliability, Cronbach’s alpha α = 0.89 [8]. The structural validity of the final version of the SF-SIS has not been assessed.

The hypothesis of unidimensionality of the SF-SIS index was assessed, performing a confirmatory factor analysis (CFA) using the results of T1. Asymptotically distribution-free estimation method was used because the items were not normally distributed. To evaluate model fit: the comparative fit index (CFI), the root mean square error of approximation (RMSEA) and the standardized root mean square residual (SRMR) were used. Following the guidelines proposed by Hu and Bentler [22] we suggest, that models with CFI ≥ 0.95, RMSEA ≤ 0.06 and SRMR ≤ 0.08 are representative of good-fitting models.

Because those results of the CFA did not support the one-factorial structure of the SF-SIS, an exploratory factor analysis (EFA) was subsequently conducted to identify the number of factors and the items loading per each factor. The suitability of the SF-SIS data for EFA was verified by using the Bartlett’s test of sphericity and the Kaiser–Meyer–Olkin measure of sampling adequacy [23]. The factors were extracted using principal axis factoring and oblique (Promax) rotation. In deciding among the various factor solutions, a scree plot of eigenvalues was considered along with the interpretability of the solution. The further, based on Guttman-Kaiser rule, the factors with eigenvalues larger than one are retained [24]. A factor loading of ≥ 0.30 was used to determine the items for each factor. The resulting factor solution was examined using CFA.

For hypotheses testing, in total five hypotheses regarding correlations between the SF-SIS and the comparative assessments as well as the discrimination between patients groups at T1 were established a priori [12]. Hypotheses one to three were based on the findings in the English pilot and validation studies reporting correlations between the SF-SIS and SIS 3.0, EQ-5D and NIHSS reaching from ρ = 0.69–0.96 [8, 9]. The DEMMI measures the construct of mobility, which is only one aspect of HRQoL measured with the SF-SIS. No studies have yet been conducted in direct comparison of these two measurement instruments. De Morton et al. [25] found correlations of the DEMMI with the Medical Outcomes Survey Short Form 36 and Assessment of Quality of Life in Elderly in Need of Care ranging from ρ = 0.17–0.50.

1. The SF-SIS will have a strong positive correlation ≥ 0.7 with the SIS 2.0.
2. The SF-SIS will have a strong positive correlation ≥ 0.7 with the EQ-5D-5L.
3. The SF-SIS will demonstrate a strong negative correlation ≥ − 0.7 with the NIHSS.
4. A weak correlation of ≥ 0.3–0.49 is assumed between the SF-SIS and the DEMMI.
5. Regarding the SF-SIS index, a statistically significant difference is expected between patients who are mildly affected (NIHSS score 0–4 points) compared to those who are moderately to severely affected (NIHSS ≥ 5 points).

### Reliability

Reliability is described as the degree to which a measurement instrument is free of measurement error [21]. In this study, the test–retest-reliability of the SF-SIS was measured by repeated measurements of the SF-SIS in unchanged patients over time [11]. Test–retest reliability was investigated in a subgroup of at least 50 subjects, reassessed one week after T1. Subjects were included, reporting no or a small (< 2 point) change in QoL on a Global Rating of Change (GRC) scale (range − 5 to + 5 points). Changes below 2 points on the GRC scale were considered not clinically relevant and thus stable QoL [26].

The Bland–Altman plot was used to visually examine the agreement of the SF-SIS’s results on the two measurement time points [27]. Homoscedastic, independent of the variable mean, and normally distributed measurement differences (T2–T1) are necessary to indicate the 95% limit of agreement. After testing for normal distribution, in addition to visually examining the distribution of measurement differences, the Kendall-tau (τ)-correlation between the absolute differences and the corresponding means was used to examine the assumption of homoscedasticity [27, 28]. A positive τ-correlation of < 0.1 is considered to represent a homoscedastic distribution [28]. Systematic differences between the two measurement time points were examined by means of a t-test for paired samples.

Test–retest reliability of the SF-SIS was calculated using the Intraclass Correlation Coefficient (ICC) with 95% confidence interval [29]. For the ICC the two-way mixed-effects model with single rater/measurement and absolute agreement was chosen [30, 31]. An ICC is given a value between 0 and 1 and an ICC ≥ 0.7 is considered acceptable [32].

Internal consistency measures the extent to which items in a scale are intercorrelated and was determined using Cronbach’s alpha coefficient [21]. Values between 0.70 and 0.95 have been proposed to indicate good internal consistency [32]. Internal consistency reliability of the SF-SIS was assessed based on the findings of the structural validity analysis.

### Responsiveness

Responsiveness is the ability of an instrument to detect change over time in the construct to be measured [21]. In order to investigate the responsiveness, four hypotheses regarding the relationship between the change scores (CS) of the SF-SIS between T1 and T3 and those of the comparative assessments were formulated a priori [33].

1. The CS of the SF-SIS will have a strong positive correlation ≥ 0.7 with the SIS 2.0’s CS.
2. The CS of the SF-SIS will demonstrate a strong positive correlation ≥ 0.7 with the EQ-5D-5L’s CS.
3. A moderate, negative correlation 0.5–0.69 is expected between the CS of the SF-SIS and the NIHSS.
4. A weak correlation of ≥ 0.3–0.49 is assumed between the CS of the SF-SIS and the DEMMI.

In addition, a GRC scale (− 5 to + 5 points) was used to record the self-assessed change in QoL. Based on these patient groups with or without a change in self-assessed QoL (GRC ≥ 2 points) the area under the receiver operator characteristics curve (AUC) was used to differentiate between subjects change scores on the SF-SIS [33]. By plotting sensitivity and 1-specificity at multiple cut-off points, the AUC can be estimated. An AUC greater than 0.7 is considered to be adequate [32].

5. The AUC is expected to be higher than 0.7 for the CS on the SF-SIS differentiating between changed and unchanged patients based on the GRC scale.

### Statistics

All analyses were carried out with SPSS 20.0 for Windows; IBM Corp.; Armonk, New York. CFA was conducted using AMOS (Version 26.0); IBM SPSS; Chicago.

The level of significance for all analyses was set at *p* < 0.05. In Interval-scaled assessments, a normal distribution of the data was checked visually using qq-plots and a statistical significance test of normality was conducted using the Shapiro–Wilk Test. For interval-scaled assessments and for normal distribution, correlations were calculated using Pearson’s correlation coefficient (r). If no normal distribution was present or for assessments with an ordinal scale level, the Spearman’s rank correlation coefficient (ρ) was used.

For comparisons between patient groups, a t-test was performed for independent samples with normal distribution (Shapiro–Wilk Test) and homogeneity of variance (Levene Test). If there was no normal distribution, the two-tailed Mann Whitney U test for independent variables was used. Missing values were reported. Single items were handled by using the calculated mean for the whole scale. In repeated measurements, missing assessments were excluded pairwise.

## Results

**Fig. 1:**
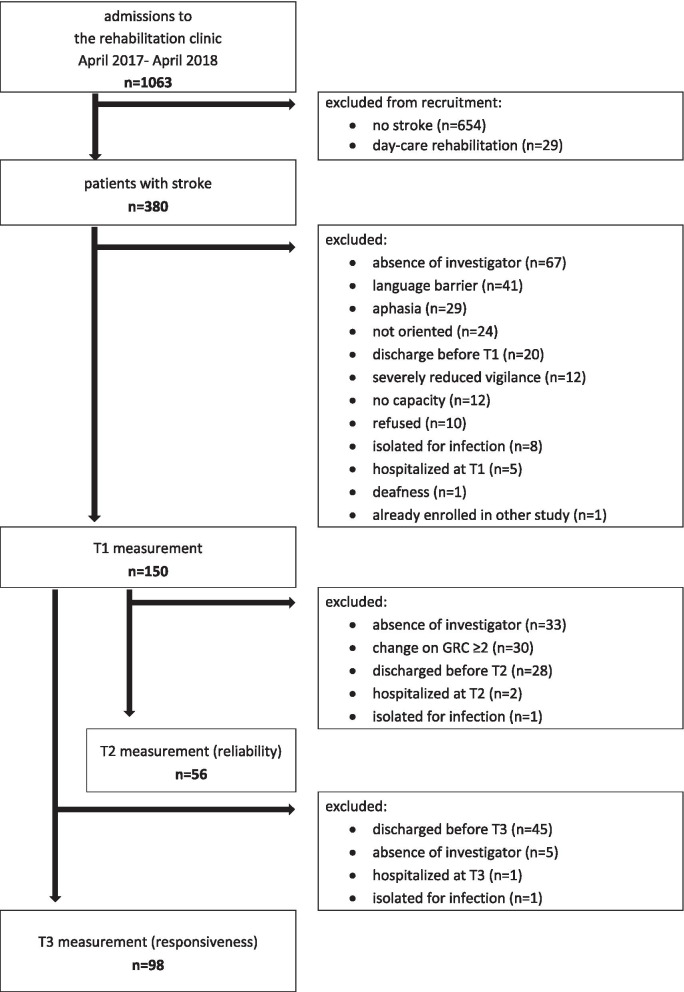
Flow chart. The flow chart shows the course of recruitment over the three measurements labelled T1 for the first appointment, T2 for the test–retest evaluation after one week and T3 at the end of inpatient rehabilitation at least two weeks after T1

Over the period of one year, 380 stroke survivors were screened for inclusion. The main reasons for exclusion were the absence of the investigator (n = 67) at the time of possible enrollment and language barriers (n = 41) (see Fig. 1). The 150 subjects included had a median age of 68 (IQR: 23) years, 38% female, and 85% had an ischemic stroke. Median duration from stroke to enrollment was 24 (IQR: 7) days (Table 1). Of the 98 patients who completed T3, two patients stopped the measurement due to exhaustion. The data collected so far were included in the analyses. For one participant, one item was missing at T3 in the SIS 2.0.

**Table 1:** Summary of sample characteristics

Measurement points	N	T1	T2	T3
		150	56	98
Age	Median (IQR) (range)	68 (23) (25–89)	73 (25) (25–89)	68 (22) (25–89)
Gender				
Male	n (%)	93 (62)	32 (57)	57 (58)
Female	n (%)	57 (38)	24 (43)	41 (42)
Primary diagnosis (ICD-10)	n (%)			
Ischemic stroke		127 (85)	47 (84)	82 (84)
Subarachnoid hemorrhage		9 (6)	4 (7)	5 (5)
Intracerebral bleeding		14 (9)	5 (9)	11 (11)
Stroke side	n (%)			
Right		70 (47)	23 (41)	44 (45)
Left		68 (45)	28 (50)	44 (45)
Bilateral		12 (8)	5 (9)	10 (10)
Number of events	n (%)			
1		134 (89)	48 (86)	86 (88)
2		16 (10)	8 (14)	12 (12)
Housing before event	n (%)			
Independent		146 (97)	54 (96)	96 (98)
Institution		4 (3)	2 (4)	2 (2)
Discharge at	n (%)			
Home		134 (89)	47 (84)	84 (86)
Institution		16 (11)	9 (16)	14 (14)
Days between event and enrollment	Median (IQR) (range)	24 (7) (14-2198)	25 (9) (14-2198)	25 (8) (14-2198)
Total duration of inpatient stay, days	M (SD) (range)	47.0 (27.6) (15-163)	52.7 (27.6) (22-163)	59.1 (26.7) (28-163)

ICD-10, International statistical classification of diseases and related health problems; IQR, interquartile range (Q3-Q1); M, mean; n, number; %, percent; SD, standard deviation

### Construct validity

On the SF-SIS, the subjects (n = 150) achieved a median of 75 (IQR: 29) points at T1. An overview of all assessment results at T1 is presented in Table 2 In all assessments, 23% of the subjects achieved values in the upper 10% of the scales.

**Table 2:** Assessment results at the first measurement point (T1)

	n	Median (IQR)	M (SD)	Range
SF-SIS	150	75 (29)	71.4 (20.0)	16–100
SIS 2.0	150	79 (25)	73.9 (18.0)	32–98
EQ-5D-5L	150	85 (26)	75.7 (21.6)	15–100
NIHSS	150	1 (3)	2.0 (2.7)	0–12
DEMMI	150	74 (28)	70.1 (24.9)	0–100

IQR, interquartile range (Q_3_–Q_1_), M, mean; n, number; SD, standard deviation

The CFA performed on the expected one-factor structure of the SF-SIS did not fit the data. Model fit indices were as follows: CFI = 0.735, RMSEA = 0.107 and SRMR = 0.150. High standardized factor loadings were observed for five out of eight items on the single factor (Table 3).

**Table 3:** Standardized factor loadings on the SF-SIS index

SF-SIS items	Estimates
Item 1	0.841
Item 2	0.472
Item 3	0.542
Item 4	0.700
Item 5	0.722
Item 6	0.786
Item 7	0.779
Item 8	0.477

**Fig. 2:**
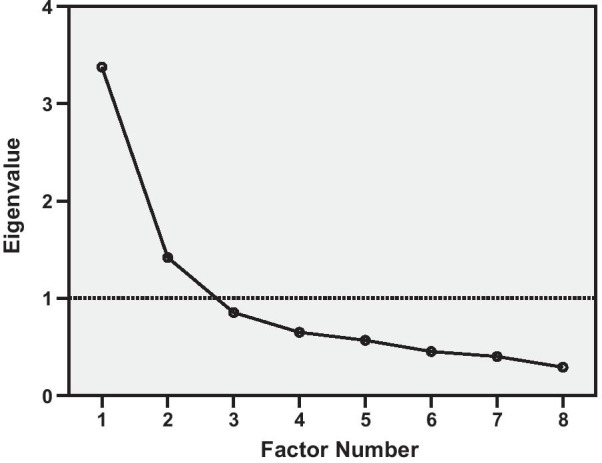
Scree plot of the SF-SIS’s eight items. The scree plot illustrates the amount of variance (y-axis) explained by the number of factors (x-axis)

Bartlett's test was highly significant (*p* < 0.001) and the Kaiser–Meyer–Olkin measure value of 0.77 supported the factorability of the matrix. The inspection of the scree plot (Fig. 2) as well as the Guttman-Kaiser rule led to the retention of two factors, accounting for 59.9% of the total variance. The one-factorial structure explained 42.2% of the variance. The inspection of the pattern matrix suggested a physical and a cognitive component of the SF-SIS (Table 4). CFA of the two-factor model showed a better but still not sufficient model fit with CFI = 0.901, RMSEA = 0.067 and SRMR = 0.084.

**Table 4:** Pattern matrix for the SF-SIS with two-factor solution

Items	Components	
	Physical	Cognitive
Item 1: Strength of leg	**0.742**	− 0.046
Item 2: Think quickly	− 0.049	**0.723**
Item 3: Have nothing to look forward to	0.099	**0.531**
Item 4: Participate in a conversation	− 0.003	**0.859**
Item 5: Do light household tasks	**0.746**	− 0.081
Item 6: Walk without losing your balance	**0.653**	0.108
Item 7: Pick up a coin	**0.732**	0.015
Item 8: Social activities	**0.367**	0.145

Bolded items indicate major loadings for each item.

Extraction method: principal axis factoring; rotation method: promax

The hypotheses regarding the correlations of the SF-SIS with the comparative assessments were evaluated by calculating Spearman's rho, due to non-normal distributed results of the assessments. Out of five hypotheses on construct validity, three hypotheses were confirmed. The correlation between the SF-SIS and the NIHSS was lower than hypothesized. The correlation between the SF-SIS and the DEMMI was higher than expected (Table 5).

**Table 5:** Correlations between assessments at the first measurement point (T1)

Correlations between SF-SIS and	Expected	Observed
SIS 2.0	≥ 0.7	0.90*
EQ-5D-5L	≥ 0.7	0.79*
NIHSS	≥ − 0.7	− 0.62*
DEMMI	0.3–0.49	0.64*

Correlations: Spearman’s rho, *p < 0.001

The fifth hypothesis regarding the discriminative validity of the SF-SIS, as one aspect of construct validity, was confirmed. Subjects who were assessed as "slightly affected" on the NIHSS (0–4 points) showed a significantly higher QoL measured with the SF-SIS in the median of 78 (IQR: 25) points (n = 131) compared to those being more severely affected (NIHSS ≥ 5, n = 19) with 41 (IQR: 22) points (median; exact Mann–Whitney U test: U = 177.5, *p* < 0.001).

### Reliability

A sample of 56 subjects was included for reliability analysis. They indicated no or a small change in the QoL (0 or 1 point) on the GRC scale. Average duration between T1 and T2 was 7.0 (SD: 0.5) days (range: 6–8 days). For the calculation of the ICC and the representation of the data in the Bland–Altman plot, a normal distribution of the measurement differences between the two points in time is necessary [34]. The given sample of n = 56 was not normally distributed (Shapiro–Wilk test). Looking at the measurement differences in the qq-plot and histogram revealed a clear outlier with a deterioration in the HRQoL on the SF-SIS of 35 points within a week, whereas the remaining data followed a normal distribution. Due to an acute depressive mood of this subject, confirmed by a psychiatric council about the hospital stay, this subject was excluded from the reliability analysis post hoc, so that 55 out of 56 subjects were analysed. The mean value of the SF-SIS at T1 was 67.1 (SD: 21.8) points, at T2 it was 72.0 (SD: 21.3) points.

**Fig. 3:**
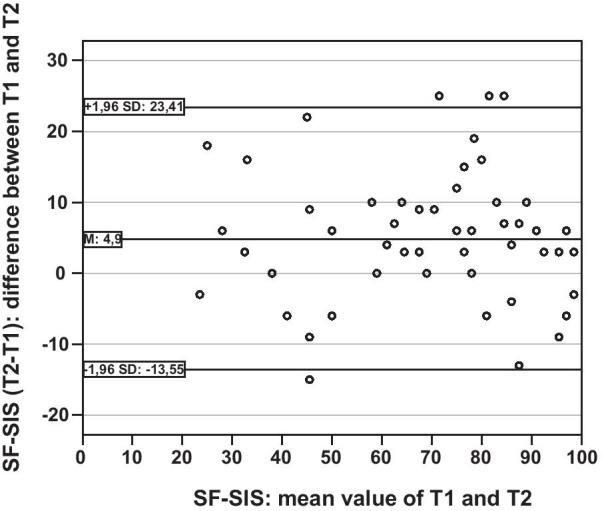
Bland–Altman plot of SF-SIS scores at two measurements in stable patients one week apart. The x-axis represents the mean scores of the two SF-SIS indices and the y-axis displays the difference between both measurements. The horizontal line in the middle visualises the mean difference between both measurements; the other two lines illustrate the 95% upper and lower limits of agreement.

The examination of the Bland–Altman plot, focusing on the measurement differences in relation to the mean differences between the two measurements, as well as a τ-correlation of 0.067 (*p* = 0.484, n = 55) suggested a homoscedastic distribution. The Bland–Altman plot can be found in Fig. 3. There was a significant measurement difference of 4.9 points (95% CI 2.4–7.5) between the two survey times of the SF-SIS (t-test for paired samples: t = 3.88, *p* < 0.001, n = 55). The lower 95% limit of agreement was − 14, the upper with a difference of 23 points on the SF-SIS. The ICC was 0.88 (95% CI 0.75–0.94). Internal consistency of the two factors identified by EFA measured with Cronbach’s alpha resulted in α = 0.79 for the physical and α = 0.75 for the cognitive domain.

### Responsiveness

All but one subject reported an improvement in QoL. The mean score on the GRC scale was 1.8 (SD: 1.8) points. On average, there were 14.8 (SD: 1.6) days between the two surveys. The SF-SIS showed an improvement in HRQoL of 8.3 (SD: 12.0) points, with a range of -22 to 44 points (Table 6). The change scores (CS) of the SF-SIS showed a normal distribution. The CS of the other assessments were not normally distributed. For this reason, Spearman’s rho was used for testing the hypotheses. The amount of the measured changes was similar in the SIS 2.0 and the EQ-5D-5L. The mean change was 6 points on these scales. The CS of all assessments can be found in Table 6.

**Table 6:** Change scores (CS) of all assessments between first and last measurement point (T3–T1)

Assessments’ CS (T3 − T1):	n	Median (IQR)	M (SD)	Range
SF-SIS	98	7 (16)	8.3 (12.0)	− 22 to 44
SIS 2.0	96	5 (10)	5.9 (8.9)	− 13 to 48
EQ-5D-5L	97	5 (10)	5.9 (12.4)	− 30 to 50
NIHSS	96	0 (1)	− 0.5 (1.0)	2 to − 4
DEMMI	96	2 (11)	5.0 (9.2)	− 15 to 38
GRC	98	2 (3)	1.8 (1.8)	− 1 to 5

IQR: interquartile range (Q_3_–Q_1_), M: mean, n: number, SD: standard deviation

None of the four hypotheses regarding the correlations of the CS of the SF-SIS with those of the comparative assessments were confirmed. The SF-SIS’s CS showed the strongest correlation with the CS of the SIS 2.0 of ρ = 0.47 (see Table 7).

**Table 7:** Correlations of assessments’ change scores (CS) between first and last measurement point (T3–T1)

Correlation between SF-SIS’s CS and CS of	Expected	Observed
SIS 2.0	≥ 0.7	0.47*
EQ-5D-5L	≥ 0.7	0.29*
NIHSS	0.5–0.69	− 0.06
DEMMI	0.3–0.49	0.26*

Correlations: Spearman’s rho, *p < 0.05

AUC was calculated to test the fifth hypothesis regarding the ability of the SF-SIS to differentiate between changed (GRC ≥ 2) and unchanged patients (GRC = 0–1) with regard to their QoL. This resulted in a value of AUC = 0.56 (95% CI 0.44–0.67). The hypothesis was therefore not confirmed. Subjects with a self-reported change in the QoL showed measurement differences between T3 and T1 on the SF-SIS of 9.5 (SD 11.5) points (n = 52) and those without a change in the QoL 6.9 (SD: 12.6) points (n = 46). A post-hoc analysis of different cut-offs on the GRC scale could not influence this indifferent result. In the post hoc analysis of the AUC for the differentiation capability of the SIS 2.0, an AUC of 0.64 (95% CI 0.53–0.75) was found.

## Discussion

In this prospective study, the psychometric quality criteria of the SF-SIS’s German version in inpatient rehabilitation were examined. Until now, only the construct validity of the SF-SIS has been investigated in the validation study of the English version [9]. The basic characteristics of the population of stroke patients included in this study are comparable to those of the English validation study. On average, the subjects were 66 years of age, with a similar gender distribution of 38% female subjects in the German and 32% in the English trial. The presence of 15% and 14% intracranial bleeding in the overall sample is also comparable [9]. The original investigation reported solely the total index value of the SIS 3.0, which was 61 (SD: 11) points [9]. The data collected for the SF-SIS in the German version showed significantly higher baseline values of 79 (IQR 25) points for the SIS 2.0 and for the SF-SIS 75 (IQR 29) points. These high values at T1, were also evident in the EQ-5D-5L with 85 (IQR 26) points and can be associated with minor limitations caused by the stroke in this population.

### Construct validity

Higher-order factor analysis of the eight domains of the SIS 3.0 by Jenkinson et al. (2013) gave them support in the derivation of a summary index for the SIS 3.0. This led to their search for a briefer tool that could be used to provide the index alone resulting in the SF-SIS [8]. The SF-SIS showed in its final version adequate face validity and acceptability for a focus group of stroke survivors and multidisciplinary stroke healthcare staff. The structural validity of the final SF-SIS had not been assessed [9].

The CFA conducted resulted in a rejection of the suggested one-factorial structure, showing lower standardized factor loadings especially of the items two and eight. The EFA of the SF-SIS resulted in a two-factor structure, a physical and a cognitive dimension, explaining 59.9% of the variance with better but still insufficient model fit in the CFA. The physical dimension included five of eight items, showing an overweight of this dimension in the total scale. The questions of the four domains of the SIS 2.0: strength, hand function, everyday life and mobility have already been summarized as a separate scale for recording physical functioning in the SIS-16 [35]. The two factors present a combination and reduction of the four factors of the SIS 3.0 suggested by Vellone et al. In their analysis a physical, cognitive, emotional and social participation factor provided better reliability and lower floor and ceiling effect than the original supposed 8-factor structure of the SIS 3.0 [36].

This is the first study that conducted the eight questions of the SF-SIS as separate questionnaire without the context of the other questions in the original eight domains of the SIS. The strong item reduction may have influenced the relevance of these items for the total index, especially more complex items might be less understandable or interpreted differently without the context of the other questions in the original SIS 2.0. We refrained from a further reduction of the items because of its brevity and the low number of items loading on each factor. Although EFA has an important role in exploring data structure, one of the disadvantages is that in practice, factors may be difficult to interpret and/ or can be inconsistent across studies [37]. For this reason, there remains a need in further analysis of the SF-SIS latent structure in a second, confirmatory, sample.

The construct validity of the SF-SIS was examined based on a priori formulated hypotheses. Three of five hypotheses, 60%, were confirmed. The correlations of the SF-SIS with the SIS 2.0 (ρ = 0.90) and the EQ-5D-5L (ρ = 0.79) were high as expected, referring to the validation study by MacIsaac et al. [9]. The relationship between the SF-SIS and the severity of the stroke measured with the NIHSS two weeks after admission to the clinic was somewhat less than expected (ρ = -0.62).

Since the SF-SIS also records items on emotions, memory, communication and participation, the strength of the correlation between mobility, measured with DEMMI, and HRQoL, measured with the SF-SIS, was expected to be only weak in the hypothesis development. In this study, the SF-SIS showed a moderate correlation with the DEMMI (ρ = 0.64). The analysis of the structural validity explains this moderate correlation between SF-SIS and mobility by a higher representation of the physical dimension in the scale. Regardless of these findings, it remains unclear whether this weighting represents an adequate reflection of the relevance of mobility for the overall construct of HRQoL in stroke survivors. The discriminative ability of the SF-SIS to differentiate between mildly and severely affected patients, recorded in inpatient rehabilitation, was confirmed.

### Reliability

The hypothesis that the SF-SIS is a reliable instrument for repeated measurements in unchanged stroke survivors has been confirmed based on the ICC and the associated 95% confidence interval with 0.88 (95% CI 0.75–0.94) [32]. A systematic measurement difference between the two time points with an improvement on the SF-SIS of 4.9 points was evident. For the interpretation of this measurement difference one has to be aware, that a change of one point on the raw score of the SF-SIS results in a change of three points on the SF-SIS Index. Nevertheless, this systematic shift in the measurement results must be taken into account, interpreting the same for repeated measurements over short time intervals. The only study that examined the test–retest reliability of the SIS 2.0, three and six months after stroke by a second survey one week later, showed ICC values of 0.57 to 0.92 for the eight domains [6]. This lack of comparative studies on the reliability of SF-SIS does not allow our results to be interpreted in a larger context. The items in the two-factors suggested by the analysis of the structural validity showed sufficiently high intercorrelations assessed by Cronach’s alpha.

### Responsiveness

Over the recorded period of 14.8 (SD: 1.6) days, the improvement in HRQoL on the SF-SIS was 8.3 (SD: 12.0) points. The CS in the comparative assessments were similar. The relatively short time between the two measurements was due to the aim of collecting the HRQoL of the patients during their inpatient stay. A priori formulated hypotheses regarding the relationship between the CS of the SF-SIS and those of the comparative assessments could not be confirmed. The correlations were significantly weaker than expected. It was conspicuous that correlations of the SF-SIS’s CS and DEMMI were comparable with the EQ-5D-5L, although the DEMMI records actually only the construct of mobility as a partial aspect of the HRQoL. Already at T1, 35% of the subjects showed no restrictions related to the stroke measured by the NIHSS and therefore only a change of one point was present. This could have influenced the NIHSS’s low correlation with the CS of the SF-SIS of only ρ = − 0.06.

Due to the SF-SIS’s inability to differentiate between self-assessed altered and unaltered subjects using the GRC scale, it cannot be recommended for use with the aim of differentiating between such groups over the determined period. The AUC for the SF-SIS was 0.56 and the visual examination of possible other cutoffs than the a priori defined change of ≥ 2 points on the GRC scale did not reveal any objective group differences. Given the slightly better differentiation capability of the SIS 2.0 (AUC = 0.64), it must be questioned whether the significant reduction of the questions to only eight in the SF-SIS could lead to an under- or overestimation of relevant changes in the HRQoL.

The use of GRC scales is always limited by the critical question of the subjects' ability to reflect the underlying construct. Assessing such a construct, in this case QoL, and quantifying possible changes over a certain period of time is a major cognitive challenge and at the same time one of the weaknesses of GRC scales [26].

Because of the lack of comparative studies, there were no reliable references in determining the strength of the expected relationships when formulating the hypotheses. For following studies, the rule of thumb recommended by Revicki et al. [38] for the correlation strength of CS between an anchor and the measuring instrument to be regarded, is considered more realistic from 0.30 to 0.35.

### Strengths and limitations

#### Population

The results of this study are based on a sufficiently large sample size of 150 subjects for validity and 55 subjects who were included in the reliability analysis [39]. For the investigation of responsiveness, 98 patients (65%) were reassessed. The discharge from rehabilitation less than two weeks after T1 was the main reason for the dropouts.

The duration between stroke and screening for inclusion was not defined, since the setting of inpatient rehabilitation should be examined. Finally, in seven percent of the patients the event was more than 100 days ago. Even in the chronic phase after a stroke, stationary rehabilitation offers, changes in functionality and also QoL changes can be expected [40, 41].

The external validity of the study is limited due to the collection of data in only one rehabilitation clinic. The recruitment took place consecutively until the previously defined sample size was reached. All patients who were admitted to the clinic were screened for inclusion and exclusion criteria. Ultimately, 150 (40%) of 380 stroke survivors were included in the study. The most common reason for exclusion had a logistical cause, due to the absence or lack of capacity of the study investigator (n = 79, 21%), who was solely responsible for the conduct of the study. Another 20 patients were discharged from the clinic before the first survey. Only ten patients (3%) did not want to participate in the study voluntarily.

The second most common reason for exclusion were non-German speaking patients (n = 41, 11%) who were not able to understand the study content and goals and thus could not give their informed consent. Another 13% (n = 53) showed cognitive or communication deficits that prevented them from participating in the study. Cognitive impairments, even years after a stroke, is a common complication [42]. For this reason, the psychometric quality criteria of a SF-SIS’s proxy version with previous cognitive screening should be evaluated in further research in order to be able to map the HRQoL of these patients as well.

#### Conduction

One strength of the data collected is the completeness of the answers. In the entire data set, there existed only one non-collected item in one questionnaire. This data quality was achieved through the recording of all assessments in personal contact and careful documentation. No incidents or adverse events occurred during the course of the study. Due to the non-normally distributed results of the recorded outcomes, the median was used to report these results.

The determination of the minimal detectable change and the minimal clinically important difference are still pending as further relevant aspects of the interpretability of the scale.

#### Conclusion

To our knowledge, this is the first prospective study to examine the psychometric quality criteria of the SF-SIS’s German version using the COSMIN checklist. Moreover, this is the first investigation of the SF-SIS’s reliability and responsiveness during inpatient rehabilitation. Comparisons of these results with the validation study of the English version are therefore limited to the examination of the convergent validity, which showed similarly high correlations with comparative measurements such as the SIS 3.0, the EQ-5D-5L and the NIHSS [9]. The analysis of structural validity did not confirm the suggested unidimensionality of the scale and found evidence for an underlying two-factor solution with a physical and cognitive domain. Further research is needed to improve and clarify the underlying factorial structure of the SF-SIS. The discriminative validity had also not been investigated so far, but showed sufficient ability to distinguish between mildly and severely affected stroke survivors. Adequate test–retest reliability was found in stable subjects after one week.

The sample included in this study was primarily only slightly affected by the stroke, which was evident in high scores in all assessments at the beginning of the survey. Examining the hypotheses on responsiveness, the strongest relationship between the CS of the SF-SIS and those of the SIS 2.0 was found, but this was below the expected strength of the hypotheses. The SF-SIS could not differentiate between patients reporting change in QoL or not over a period of about two weeks. It has to be emphasized, that the responsiveness of the SIS, although being in use internationally for a long time, has not been adequately investigated and therefore no comparisons can be drawn. An examination of whether changes on the SF-SIS, over longer periods such as months to years after the event, could more adequately reflect the perceived changes in the patient would be desirable for the SF-SIS’s use beyond the inpatient setting.

## Data Availability

The datasets used and/or analysed during the current study are available from the corresponding author on reasonable request.

## Abbreviations

AUC: Area under the receiver operating characteristic curve
CFA: Confirmatory factor analysis
CFI: Comparative fit index
CI: Confidence interval
COSMIN: COnsensus-based Standards for the selection of health Measurement Instruments
CS: Change score
DEMMI: De Morton Mobility Index
EFA: Exploratory factor analysis
GRC: Global rating of change
HRQoL: Health-related quality of life
ICC: Intraclass correlation coefficient
ICD-10: International statistical classification of diseases and related health problems
Mean (SD): Mean (standard deviation)
Median (IQR): Median [interquartile range (Q3-Q1)]
NIHSS: National Institutes of Health Stroke Scale
QoL: Quality of life
RMSEA: Root mean square error of approximation
SD: Standard deviation
SF-SIS: Short form of the Stroke Impact Scale
SIS: Stroke Impact Scale
SRMR: Standardized root mean square residual
VAS: Visual analog scale
VISTA: Virtual International Stroke Trials Archive
